# A Prospective Population-Based Study of Cardiovascular Disease Mortality following Treatment for Breast Cancer among Men in the United States, 2000–2019

**DOI:** 10.3390/curroncol30010023

**Published:** 2022-12-25

**Authors:** Duke Appiah, Megan Mai, Kanak Parmar

**Affiliations:** 1 Department of Public Health, Texas Tech University Health Sciences Center, Lubbock, TX 79430, USA; 2 School of Medicine, Texas Tech University Health Sciences Center, Lubbock, TX 79430, USA; 3 Department of Internal Medicine, Texas Tech University Health Sciences Center, Lubbock, TX 79430, USA

**Keywords:** breast cancer, cardiovascular disease, chemotherapy, radiotherapy, mortality

## Abstract

Male breast cancer is rare but its incidence and mortality are increasing in the United States, with racial/ethnic disparities in survival reported. There is limited evidence for cardiotoxicity of cancer treatment among men with breast cancer. We evaluated the relation between breast cancer treatment and cardiovascular disease (CVD) mortality among men and investigated the salient roles that race/ethnicity play on this relation. Data were from 5216 men with breast cancer aged ≥ 40 years from the Surveillance, Epidemiology, and End Results program who were diagnosed from 2000 to 2019 and underwent surgery. Competing risk models were used to estimate hazards ratios (HR) and 95% confidence intervals (CI). During a median follow-up of 5.6 years, 1914 deaths occurred with 25% attributable to CVD. In multivariable-adjusted models, men who received chemotherapy had elevated risk for CVD (HR: 1.55, 95%CI: 1.18–2.04). This risk was higher among Hispanic men (HR: 3.96, 95%CI: 1.31–12.02) than non-Hispanic Black and non-Hispanic White men. There was no significant association between radiotherapy and CVD deaths. In this population-based study, treatment with chemotherapy was associated with elevated risk of CVD mortality in men with breast cancer. Racial/ethnic disparities in the association of chemotherapy and CVD mortality were observed.

## 1. Introduction

Male breast cancer (MBC) is a rare and understudied cancer that accounts for about 1% of all breast cancer cases in the United States [1]. Over the past few decades, the incidence of MBC has been on the rise. In 2022, it was estimated that 2710 new cases and 530 deaths from MBC will occur, representing an increase of 94% and 33%, respectively from estimates for 2000 [2,3]. While substantial efforts have been made in the past 20 years to understand the biologic features, effective treatment modalities, and outcomes for breast cancer, MBC remains largely understudied compared to female breast cancer (FBC) [4,5].

Due to its rarity, most MBC patients in the past have not been included in therapeutic studies, therefore, treatment strategies for MBC have largely been extrapolated from evidence from FBC patients [4,5,6,7]. While MBC share similar characteristics with FBC, it has distinct features that may influence treatment outcomes [1,8]. For example, MBC usually occurs at older ages and approximately occur 5 years before FBC [1]. While young MBC patients tend to have better overall survival than older male patients diagnosed with breast cancer, young MBC patients have worse survival outcomes than young FBC patients [9]. The lack of established screening guidelines for breast cancer in men often results in delays in the diagnosis of MBC of about 21 months after the onset of symptoms [6]. Furthermore, MBC patients are less likely to receive conventional treatments that may partly be due to low compliance among MBC patients [10]. Other clinicopathological differences include MBC patients having higher frequency of mutations in BRCA2 tumor suppressor gene compared to BRCA1, having more frequent lymph node metastases, and having a higher proportion of estrogen-receptor positive tumors [1,5,6]. 

Although inconclusive, several studies have reported worse prognosis for MBC patients compared to FBC patients [4]. Recent registry-based studies have reported lower overall and 5-year survival in MBC compared to FBC patients, with the risk of death in MBC patients being 19% to 43% higher than FBC after controlling for potential confounding factors [4,10]. Clinical characteristics and undertreatments are reported to explain about 63% of the excess mortality for MBC patients [10]. Among MBC patients, racial and ethnic disparities in survival and other clinicopathological characteristics have also been reported with racial and ethnic minority men having lower overall survival compared to non-Hispanic White men [11].

Noncancer death, especially cardiovascular disease (CVD)-related deaths, accounts for a large proportion of deaths in MBC survivors as these two conditions share in common several risk factors [6,12]. Only a few studies have evaluated CVD outcomes in MBC patients [6,12,13]. A recent population-based epidemiologic study reported higher CVD mortality among MBC patients than would have been expected compared to the general population, with the mortality being highest among younger MBC patients aged 35–44 years at diagnosis [6]. 

Tremendous changes in treatment modalities for breast cancer have occurred over the past five decades that has been suggested to influence cardiovascular outcomes among breast cancer survivors [5,14,15]. The etiology of cardiotoxicity has been reported to vary by the type of cancer therapy. For example, HER2-directed therapeutics and chemotherapeutics such as anthracyclines that are standard-of-care treatment in high-risk individuals have been reported to increase the risk for cardiomyopathy and heart failure [16,17]. However, there is limited evidence on the pertinent roles that treatment for breast cancer plays on CVD outcomes in men. Recent ASCO guidelines on MBC recommend conducting post-treatment surveillance studies to provide evidence-based data for the management of breast cancer in men [18]. Therefore, the primary aim of this study was to evaluate the relation between breast cancer treatment and CVD mortality among men in the United States. The secondary aim was to investigate racial and ethnic disparities in the relation of breast cancer treatment and CVD mortality. 

## 2. Materials and Methods

### 2.1. Study Population

Data for this registry-based prospective cohort study were obtained from National Cancer Institute's Surveillance, Epidemiology, and End Results (SEER) program which covers about 34% of the US population and obtains information from 17 population-based cancer registries located in the following states: Alaska, California, Connecticut, Georgia, Hawaii, Iowa, Kentucky, Louisiana, New Mexico, New Jersey, Utah. and Washington [19]. Men aged ≥ 40 years who were diagnosed with histologically confirmed stage I–III primary breast cancer from 1 January 2000 to 31 December 2019 were eligible for the current study. Of the 5508 eligible samples whose diagnosis was not made only at autopsy or via death certificates, the following exclusions were made: 219 persons that did not undergo surgery, 72 persons with no follow-up information or unknown cause of death, and 1 person with no information on tumor laterality. This resulted in an analytic sample of 5216 MBC patients. Institutional review board approval was not required for this study as the SEER registry is a de-identified publicly available database. 

### 2.2. Definition of Study Variables

Information obtained from the SEER database included age at diagnosis, year of diagnosis, race and ethnicity, geographic region, location (rural or urban), marital status, annual median household income of the county of patient’s residence, disease stage, tumor grade, tumor size, laterality, number of regional lymph nodes examined, hormone (estrogen and progesterone) receptor status, cancer therapy, cause of death, and survival time. 

The main exposure of interest in the current study was first course of cancer therapy. For the current analysis, both radiotherapy and chemotherapy were classified as received or not received. The reported sensitivity, specificity, and positive predictive value of the SEER database correctly identifying individuals with breast cancer who received therapy are 69%, 98%, and 91% for chemotherapy and 80%, 98%, and 98% for radiotherapy [20]. Breast cancer was defined using International Classification of Diseases for Oncology, 3rd edition (ICD-O-3) codes C500-C509. Race and ethnicity were defined as non-Hispanic White, non-Hispanic Black, Hispanic, and other which includes American Indians/Alaska Native, Asian or Pacific Islander, and other race or ethnic groups. Cancer stage at time of diagnosis was defined using the American Joint Committee on Cancer’s staging manual that uses information on tumor size, regional lymph node involvement, and the presence of metastasis [21]. Editions of the manual that were applicable during the period of the current study were used. 

Cause of death information was classified using the World Health Organization’s International Classification of Diseases, Tenth Revision codes. CVD mortality was defined as deaths due to diseases of heart (I00–I09, I11, I13, I20–I51), hypertensive heart disease (I10–115), cerebrovascular diseases (I60–I69), atherosclerosis (I70), aortic aneurysm and dissection (I71), or other diseases of arteries, arterioles, or capillaries (I72–I78). 

### 2.3. Statistical Analysis

Characteristics of men at the time of cancer diagnosis were described and compared among cancer treatment groups using chi-square test. Competing risk analyses were conducted using cause-specific hazard models, with deaths from all other causes besides CVD considered as competing risk events. Because the incidence of MBC and CVD mortality rates increase with advancing age, attained age in years was used as the time scale for all time-to-event analyses. Thus, estimates from such model are age-adjusted [22]. The validity of the proportional hazards assumption was tested and confirmed using weighted Schoenfeld residuals as well as using formal statistical test of non-proportionality. 

Covariate selection for multivariable models was based on variables that were significant in bivariable analyses at an alpha of 0.2. Variables evaluated in bivariate models were year of diagnosis, race and ethnicity, geographic region, location, marital status, income, disease stage, estrogen and progesterone receptor status, tumor grade, tumor size, laterality, and number of regional lymph nodes examined. Because the impact of radiation on overall survival among MBC patients is not the same between breast conservation surgery and mastectomy [23], and radiation to the left side of the breast is associated with a higher risk of CVD than on the right breast [24], additional analyses were performed to evaluate the role of type of surgery and tumor laterality on the association of radiotherapy with CVD mortality among men who did not receive chemotherapy.

Finally, interaction between race and ethnicity with cancer therapy was tested. The SEER*Stat version 8.4.0.1 software (Information Management Systems, Rockville, MD, USA) and the SAS software version 9.4 (SAS Institute, Inc., Cary, NC, USA) were used to conduct the statistical analyses with statistical significance determined with a two-tailed test *p* value of less than 0.05.

## 3. Results

Over the study period, there was an increase in the number men diagnosed with cancer. Approximately 20% of men with breast cancer were diagnosed in 2000–2004 compared to 28% in 2015–2019. The mean age at diagnosis was 66.1 (standard deviation: 11.7; median 66) years, with more than half of them (52%) living in the west region of the United States at the time of cancer diagnosis. The racial and ethnic distribution of the sample are as follows: non-Hispanic White, 75%; non-Hispanic Black, 12%; and Hispanic, 7%. Only 12% of men diagnosed with breast cancer lived in counties with median household incomes of less than $50,000. With regards to receipt of cancer treatment, 38.8% and 28.4% of men reported receiving chemotherapy and radiotherapy, respectively, with the median time from diagnosis to treatment being 1 month. Characteristics of participants according to the first course of cancer therapy received are reported in Table 1. 

Over the period of the study, the proportion of patients who received radiotherapy (with or without chemotherapy) increased while the proportion of patients who received chemotherapy reduced with age. Additionally, a greater proportion of patients who received both chemotherapy and radiotherapy had stage III cancer while the proportion of patients with mastectomy was lowest among those who received radiotherapy alone. 

During a median follow-up of 5.6 years (interquartile range: 2.6 to 9.8), 1914 deaths occurred with 25% and 35% attributable to CVD and breast cancer, respectively. Characteristics of patients at the time of diagnosis according to cardiovascular disease mortality status are presented in Table 2. Of the 485 CVD deaths, 64.5% occurred among patients who received neither chemotherapy or radiation, 14.6% occurred among those who received chemotherapy but not radiation, 11.8% occurred among patients who received radiation but not chemotherapy, and 9.1% occurred among patients who received both chemotherapy and radiotherapy. Similarly, among the 1914 all-cause mortality cases, 55.7% occurred among patients who received neither chemotherapy nor radiation, 19.1% occurred among those who received chemotherapy but not radiation, 10.2% occurred among patients who received radiation but not chemotherapy, and 15.0% occurred among patients who received both chemotherapy and radiotherapy.

Multivariable models were adjusted for age, year of cancer diagnosis, race and ethnicity, disease stage, tumor size, and number of lymph nodes examined as these variables were found to be statistically significant in bivariable analyses. In these models, men with breast cancer who received chemotherapy as part of their first course of treatment had elevated risk for CVD (Hazard ratio (HR): 1.32, 95% CI: 1.05–1.66)), with the risk being higher among those who received chemotherapy alone (HR: 1.55, 95% CI: 1.18–2.04) (Table 3). 

**Figure 1:**
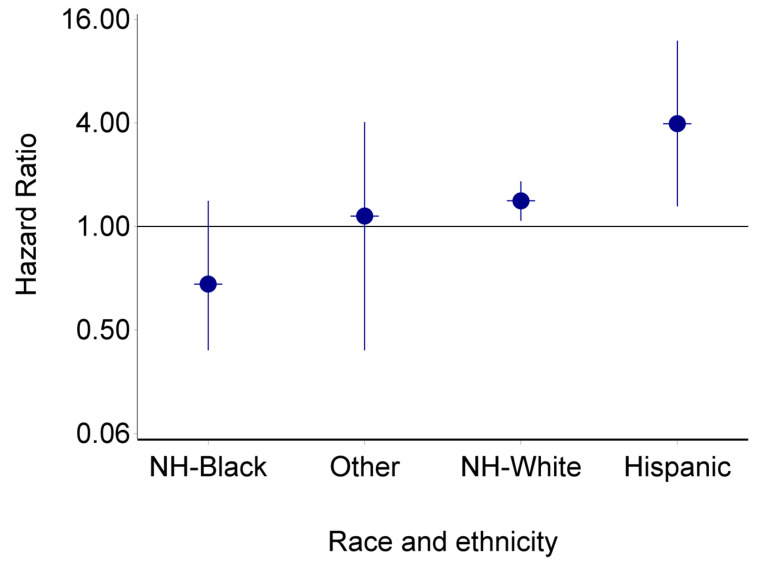
The association of cancer treatment with cardiovascular disease mortality in men diagnosed with breast cancer according to race and ethnicity, SEER registry (2000–2019). NH: Non-Hispanic. *p* value for interaction = 0.005.

There was no significant association between radiotherapy (with or without chemotherapy) and CVD deaths. There was a significant interaction between race and ethnicity and cancer treatment on the risk of CVD mortality (*p* = 0.005). The risk of CVD mortality was observed to be highest among Hispanic men (HR: 3.96, 95% CI: 1.31–12.02) (Figure 1). 

Among persons who received radiotherapy, there was no significant influence of laterality or the association of radiotherapy and CVD mortality (*p* = 0.672). Similarly, the relation of radiotherapy and CVD mortality was not significantly influenced by the type of surgery, thus breast conservation surgery or mastectomy (*p* = 0.206).

## 4. Discussion

In this population-based study of men diagnosed with breast cancer in the United States over a 20-year period, treatment with chemotherapy was associated with elevated risk of CVD mortality, while no significant association was observed between radiation therapy and deaths due to CVD. Racial and ethnic disparities in the association of chemotherapy and CVD mortality were observed, with Hispanic men having higher risk of CVD deaths compared to non-Hispanic Black and non-Hispanic White men. To our knowledge, this is the first study to comprehensively characterize CVD mortality due to cancer treatment among men diagnosed with breast cancer.

Some breast cancer therapeutics have been reported to result in early or delayed cardiotoxicity comprising of hypertension, arrhythmias, pericarditis, thromboembolism, valvular disease, left ventricular dysfunction, heart failure, and myocardial infarction [14,15]. Accordingly, it has been estimated that the cumulative incidence of treatment-related cardiotoxic outcomes among breast cancer patients may be as high as 33% [25]. There are limited prospective investigations of the relation of neoadjuvant or adjuvant chemotherapy on CVD morality in MBC patients. Results from the current study of elevated risk of CVD mortality among MBC patients who received chemotherapy is supported by several pieces of evidence of the cardiotoxic effects of chemotherapy in murine models and studies conducted among FBC patients [14,26,27,28,29,30]. The most widely reported cardiotoxic effect of chemotherapy is left ventricular dysfunction that manifests as overt heart failure over time [14,25], although other cardiac events such as thrombosis, arrhythmias, myocarditis, pericarditis, and myocardial infarction have also been reported [31]. For instance, Yang et al. [32] reported a 74% elevated risk of heart failure among breast cancer patients who received chemotherapy. Conversely, as seen in some studies among women [33], a few studies conducted mostly among small samples of men with breast cancer have also reported lower mortality in men who received adjuvant chemotherapy [34,35,36]. However, these studies did not specifically evaluate cardiovascular-related mortality. 

There are several mechanisms by which chemotherapy may influence cardiovascular health in breast cancer patients. Anthracyclines, such as doxorubicin interacts with deoxyribonucleic acids, intercalating and inhibiting macromolecular biosynthesis of cardiac myocytes that eventually leads to apoptosis of myocytes and permanent damage to the myocardium [14,31]. Additionally, chemotherapeutics fosters the generation of reactive oxygen species which damage deoxyribonucleic acids, proteins, and mitochondrial membrane of myocytes [14,31]. In light of this, finding avenues to reduce the risk of CVD events among MBC patients is of great importance. With adjuvant chemotherapy not improving overall or breast cancer-specific survival among MBC patients with stage I and IIA cancer, the risk of CVD mortality may be reduced in this population by perhaps skipping chemotherapy for MBC patients with early-stage disease [37]. In addition, more consideration may be given to administering adjuvant trastuzumab which often, but not always, results in reversible LV dysfunction together with chemotherapy for patients with HER2-positive early-stage breast due to the reported marked improvement in survival and reoccurrence of cancer with this treatment regimen [14,38]. Finally, the risk-benefit profile of each MBC patient should be taken into consideration when choosing chemotherapy especially for those who are at high risk for CVD [14]. For those who have a risk-benefit profile in favor of chemotherapy, early detection and interception of cardiotoxicity remains important for clinicians.

Emerging evidence suggests that there are declining CVD mortality trends by radiation therapy among breast cancer patients [16,39]. Vo et al. [16] evaluating trends in heart disease mortality in the United States among women with invasive breast cancer from 1975 to 2017 observed significant declines in heart disease mortality for breast cancer survivors treated with radiotherapy alone compared to the general population, while an increasing trend in heart disease mortality was seen for regional stage patients treated with chemotherapy alone. From 1975–1984 to 2005–2016, the 10-year cumulative heart disease mortality declined from 6.35% to 2.94% among breast cancer survivors treated with radiotherapy alone while the 10-year cumulative heart disease mortality reduced from 1.78% to 1.21% [16]. Similarly, Hooning et al. [39] studying 7425 patients in the Netherlands treated for early breast cancer from 1970 to 1986 and followed through to 2000 found no increased CVD mortality for post-lumpectomy radiation, with the risk estimates for CVD mortality highest for post-lumpectomy radiation administered before 1979. Studies conducted in the modern era of breast cancer therapy have largely found no association between radiation therapy and CVD outcomes [31,39]. Similar to the current study where no association between radiotherapy (with or without chemotherapy) and CVD mortality, regardless of tumor laterality, was observed among MBC patients, Onwudiwe et al. using data from women aged 66 years and older with stage 0–III breast cancer diagnosed between 2000 and 2005 in the SEER-Medicare database also observed no association between radiation therapy and combined endpoints of death or cardiovascular disease [40]. Another register-based matched cohort study of Swedish breast cancer patients diagnosed from 2001 to 2008 and followed up until 2017 also observed no elevated risk of heart disease following locoregional radiotherapy [32].

The lack of a positive association of radiotherapy with CVD mortality observed in the current study as well as other studies of cancer therapy administered in the 21st century reflects the impact of changes in radiotherapy procedures [5,14]. However, it should be noted that radiation-associated cardiotoxicity often appears about 10 to 30 years after treatment and most studies including the current study did not have any individuals with follow-up beyond 20 years [41]. Increasing clinical guidelines about the adverse cardiac effects of radiation therapy has advanced cardio-protection strategies to minimize radiation-related damage to the cardiovascular system [14]. For example, reduction in radiation doses to the left side of the chest during radiotherapy, positioning patients to displace the heart during radiotherapy administration, the use of more precise radiotherapy using imaging and brachytherapy, and alternative radiotherapy options have all gone a long way to reduce the effects of radiation therapy on cardiac damage during cancer treatment [16,32,42,43,44,45]. Alternatively, the null association between radiotherapy and CVD mortality in MBC patients may be due to patients with left-sided breast cancer being less likely to be selected for radiotherapy due to the proximity of the tumor to the heart [32,45]. Future studies evaluating dosages of radiation to the heart and CVD mortality will enhance our understanding of a safe threshold of radiation that enhances cancer treatment response and at the same time reduce the risk for CVD outcomes in breast cancer patients.

Another interesting observation from the current study is the racial and ethnic disparities in the relation of chemotherapy with CVD mortality in MBC patients. The risk of CVD mortality in Hispanic men was more than twice the risk in non-Hispanic White men with no association observed between chemotherapy and CVD mortality among non-Hispanic Black men with breast cancer. While reasons for these findings are largely unknown, it is possible that differences in sociodemographic, socioeconomic, behavioral, and biological factors as well as differences in access to cancer treatment may partly explain these findings. For instance, compared to non-Hispanic White individuals, Hispanic populations are less likely to partake in mammography screening and adhere to cancer screening recommendations [46,47,48]. Thus, they often experience longer times to diagnosis of cancer resulting in them being likely to be diagnosed with advanced staged cancer [46]. Furthermore, they often experience poor quality of life following diagnosis of cancer than non-Hispanic White individuals [46]. Due to language barriers among low-acculturated Hispanic individuals, they often receive limited communication about cancer diagnosis and treatment which hinders the decision-making processes concerning cancer treatments [46,49]. A few studies among men [50] and several studies among women with breast cancer consistently report longer delays in receipt of chemotherapy among Hispanic and non-Hispanic Black individuals [51,52,53]. Taken together, it is possible that all these factors may contribute to the high risk of CVD mortality due to chemotherapy among Hispanic population. 

With non-Hispanic Black individuals also experiencing delays in chemotherapy treatment [54,55] despite rates of oncologic consultation being similar between Black and White cancer patients [56], it would have been expected that this population would also experience high CVD risk due to chemotherapy. However, this was not the case in the current study. We speculate that the greater proportion of early discontinuation of chemotherapy of non-Hispanic Black patients mostly due to negative beliefs about efficacy of chemotherapy often due to concern about adverse effects [55,57,58], coupled with Black patients having lower pathologic complete response to neoadjuvant chemotherapy than Hispanic and other racial groups [59] may result in them having reduced exposure to the cardiotoxic effect of chemotherapeutics. With delays and interruptions in breast cancer treatment being positively related to breast cancer-specific mortality [60], this explanation is further supported by the observation that Black MBC patients have greater breast cancer-related mortality than CVD mortality compared to MBC patients of other racial and ethnic groups [12,50,61]. 

Currently, most treatment options for breast cancer in men are based on evidence from trials among women with breast cancer [62]. Although some reports show that treatment options in men produce comparable results to FBC patients [63], overall survival in MBC patients is lower than those for FBC patients [64] with some studies reporting excess mortality rates of about 60% in men when compared to women [10]. The lack of evidence-based treatment recommendations and screening guidelines for breast cancer in men, coupled with limited reports on treatment-associated complications continue to impact treatment choices and care for men with breast cancer [62]. Some studies report that screening mammography yields similar cancer detection rates between men and women at high risk for breast cancer [65]. Therefore, interventions focusing on increasing awareness and promoting breast cancer education in men, together with enhancing access to care among high-risk groups regardless of race and ethnicity will go a long way to increase early-stage cancer diagnosis and reduce racial and ethnic disparities in survival outcomes [66]. Furthermore, the few clinical trials among male breast cancer patients [18,67,68] currently underway will provide comprehensive data on the long-term management of MBC to inform treatment recommendations and guidelines on regimens that optimize cancer therapy and at the same time limit the risk of CVD [15]. 

The strength of the current study includes the use of a large population-based sample of MBC patients selected within a modern timeframe of cancer treatment. Limitations of the study include the lack of detailed information on specific drugs or hormone therapy not being available in the SEER registry for most of the period of observation for this study. HER2 positivity status was not evaluated in the current study as such information was only available after 2010. Furthermore, information on CVD risk factors at the time of cancer diagnosis as well as information on other comorbid noncancer diseases were not collected by SEER program. Finally, the chance of misclassification bias influencing the results of the study due to the use of death certificates to identify deaths attributable to CVD cannot be entirely ruled out. However, cause-of-death information in the SEER registry have been reported to have good validity [69].

## 5. Conclusions

In this population-based study of men with breast cancer, treatment with chemotherapy was significantly associated with elevated risk of CVD mortality, with the highest risk observed among Hispanic men. These findings have important implications for cardio-oncology care as well as extending research in the context of noncancerous outcomes in men with breast cancer. With the proportion of cancer patients receiving radiation therapy and chemotherapy increasing over the past few decades [16], future studies on cardiovascular outcomes due to cancer treatment regimens among racially and ethnically diverse MBC patients are warranted to enhance the clinical management of breast cancer in men. 

## Figures and Tables

**Table 1:** Characteristics of men at the time of breast cancer diagnosis according to cancer therapy, SEER program (*n* = 5216).

	Cancer Treatment Groups				
Characteristics, %	No Chemotherapy, No Radiotherapy (*n* = 2653)	Chemotherapy, No Radiotherapy (*n* = 1083)	No Chemotherapy, Radiotherapy (*n* = 541)	Chemotherapy and Radiotherapy (*n* = 939)	*p* Value
Age, years					<0.001
40–64	34.7	61.6	34.2	60.7	
65–74	30.5	29.1	29.6	30.8	
≥75	34.8	9.3	36.2	8.5	
Year of diagnosis					<0.001
2000–2004	22.2	20.4	17.2	19.5	
2005–2009	24.3	26.5	18.9	20.9	
2010–2014	26.8	28.7	28.5	26.2	
2015–2019	26.7	24.4	35.5	33.4	
Race and ethnicity					0.447
Non-Hispanic White	76.10	73.80	74.70	73.80	
Non-Hispanic Black	11.70	12.20	12.60	13.50	
Hispanic	6.50	7.70	8.50	7.00	
Other	5.70	6.40	4.30	5.60	
Region					0.003
Midwest	4.3	3.9	4.6	5.1	
Northeast	20.0	21.9	20.9	16.5	
South	20.8	23.7	24.2	26.6	
West	54.9	50.5	50.3	51.8	
Marital status, married	68.5	69.3	68.2	69.0	0.947
Median household income					0.992
<$50,000	12.2	12.2	12.4	12.8	
$50,000–$75,000	54.2	53.2	53.0	53.7	
>$75,000	33.5	34.6	34.6	33.5	
Location, rural	12.6	13.2	9.2	11.5	0.103
Stage					<0.001
I	51.5	27.1	41.6	11.9	
II	41.0	54.8	39.7	41.7	
III	7.6	18.1	18.7	46.3	
Tumor grade					<0.001
I/II	68.7	53.70	65.10	54.10	
III/IV	24.6	42.50	27.70	42.20	
Unknown	6.7	3.8	7.2	3.7	
Lymph nodes examined					<0.001
0	9.1	2.0	7.6	1.8	
≥1	90.9	98.0	92.4	98.2	
Tumor size (cm)					<0.001
<2	46.3	35.6	44.4	26.9	
≥2	35.3	48.0	41.4	57.1	
Unknown	18.4	16.3	14.2	16.0	
ER status					<0.001
Yes	89.9	91.7	94.5	92.8	
No	1.9	3.7	2.0	4.8	
Unknown	8.1	4.6	3.5	2.4	
PR status					<0.001
Yes	82.9	80.0	89.1	82.9	
No	7.5	13.7	6.7	14.2	
Unknown	9.6	6.4	4.3	3.0	
Type of surgery					<0.001
Breast conservation surgery	9.4	7.1	35.2	12.2	
Mastectomy	90.6	92.9	64.8	87.8	

ER: estrogen receptor, PR: progesterone receptor status.

**Table 2:** Characteristics of men at the time of breast cancer diagnosis according to cardiovascular disease mortality status at the end of follow-up, SEER registry (*n* = 5216).

Characteristics, %	CVD Mortality		*p* Value
	No (*n* = 4731)	Yes (*n* = 485)	
Age, years			<0.001
40–64	47.6	18.6	
65–74	30.4	27.6	
≥75	22.0	53.8	
Year of diagnosis			<0.001
2000–2004	19.2	37.1	
2005–2009	22.5	34.0	
2010–2014	27.9	20.6	
2015–2019	30.4	8.2	
Race and ethnicity			0.087
Non-Hispanic White	74.6	79.4	
Non-Hispanic Black	12.4	10.7	
Hispanic	7.1	6.2	
Other	5.9	3.7	
Region			0.002
Midwest	4.2	6.2	
Northeast	20.1	17.3	
South	23.4	17.7	
West	52.3	58.8	
Marital status, married	69.0	66.2	0.217
Median household income			0.825
<$50,000	12.4	11.5	
$50,000–$75,000	53.7	54.8	
>$75,000	33.9	33.6	
Location, rural	12.1	12.8	0.662
Stage			0.001
I	39.0	30.5	
II	43.1	51.5	
III	17.9	17.9	
Tumor grade			0.780
I/II	62.7	61.4	
III/IV	31.7	33.2	
Unknown	5.6	5.4	
Lymph nodes examined			<0.001
0	5.4	13.4	
≥1	94.6	86.6	
Tumor size (mm)			<0.001
<2	41.4	30.5	
≥2	42.7	40.4	
Unknown	15.9	29.1	
ER status			<0.001
Yes	91.6	88.0	
No	3.0	1.4	
Unknown	5.4	10.5	
PR status			<0.001
Yes	83.2	80.6	
No	10.1	7.4	
Unknown	6.7	12	
Type of surgery			0.380
Breast conservation surgery	12.2	10.8	
Mastectomy	87.8	89.2	
Cancer therapy			<0.001
No chemotherapy, no radiotherapy	49.5	64.5	
Chemotherapy, no radiation	21.4	14.6	
Radiation, no chemotherapy	10.2	11.8	
Chemotherapy and radiotherapy	18.9	9.1	

ER: estrogen receptor, PR: progesterone receptor status.

**Table 3:** Hazard ratios and 95% confidence intervals for the association of cancer treatment with CVD mortality in men diagnosed with breast cancer, SEER registry (2000–2019).

Treatment	Model 1		Model 2	
	HR (95% CI)	*p* Value	HR (95% CI)	*p* Value
Chemotherapy		<0.001		0.019
No	1		1	
Yes	1.56 (1.25–1.94)		1.32 (1.05–1.66)	
Radiotherapy		0.385		0.848
No	1		1	
Yes	1.10 (0.88–1.38)		0.98 (0.77–1.24)	
Radiotherapy and/or chemotherapy		<0.001		0.018
No chemotherapy or radiotherapy	1		1	
No radiotherapy, chemotherapy	1.80 (1.38–2.35)		1.55 (1.18–2.04)	
Radiotherapy, no chemotherapy	1.15 (0.86–1.52)		1.08 (0.81–1.45)	
Radiotherapy and chemotherapy	1.34 (0.97–1.85)		1.07 (0.76–1.52)	

Model 1: age adjusted model. Model 2: adjusted for age, year of cancer diagnosis, race and ethnicity, disease stage, tumor size, and number of lymph nodes examined. CI: confidence interval, HR: hazard ratio.

## Data Availability

Data used for this study are publicly available from the National Cancer Institute at https://seer.cancer.gov/, accessed on 20 November 2022.
